# Prefrontal GABA Levels Correlate with Memory in Older Adults at High Risk for Alzheimer’s Disease

**DOI:** 10.1093/texcom/tgaa022

**Published:** 2020-06-08

**Authors:** Geetanjali Murari, Darren Ri-Sheng Liang, Aliya Ali, Frankie Chan, Mirjam Mulder-Heijstra, Nicolaas Paul L G Verhoeff, Nathan Herrmann, J Jean Chen, Linda Mah

**Affiliations:** Rotman Research Institute, Baycrest Health Sciences, Toronto, ON M6A 2E1, Canada; Rotman Research Institute, Baycrest Health Sciences, Toronto, ON M6A 2E1, Canada; Rotman Research Institute, Baycrest Health Sciences, Toronto, ON M6A 2E1, Canada; Rotman Research Institute, Baycrest Health Sciences, Toronto, ON M6A 2E1, Canada; Rotman Research Institute, Baycrest Health Sciences, Toronto, ON M6A 2E1, Canada; Department of Psychiatry, Geriatric Psychiatry Division, University of Toronto, Toronto, ON M5T 1R8, Canada; Department of Psychiatry, Baycrest Health Sciences, Toronto, ON M6A 2E1, Canada; Department of Psychiatry, Geriatric Psychiatry Division, University of Toronto, Toronto, ON M5T 1R8, Canada; Sunnybrook Research Institute, Sunnybrook Health Sciences Centre, Toronto, ON M4N 3M5, Canada; Rotman Research Institute, Baycrest Health Sciences, Toronto, ON M6A 2E1, Canada; Department of Medical Biophysics, University of Toronto, Toronto, ON M5G 1L7, Canada; Rotman Research Institute, Baycrest Health Sciences, Toronto, ON M6A 2E1, Canada; Department of Psychiatry, Geriatric Psychiatry Division, University of Toronto, Toronto, ON M5T 1R8, Canada

**Keywords:** γ-aminobutyric acid, cognition, magnetic resonance spectroscopy, nonverbal memory, verbal memory

## Abstract

γ-Aminobutyric acid (GABA), a primary inhibitory neurotransmitter in the brain, plays a significant role in aging and in neurodegenerative disorders, including Alzheimer’s disease (AD). We investigated the relationship between GABA levels in the dorsomedial/dorsoanterolateral prefrontal cortex (DM/DA-PFC) and memory in high-AD risk participants. Thirty-eight participants (14 Cognitively Normal [CN], 11 with Subjective Cognitive Decline (SCD), and 13 Mild Cognitive Impairment [MCI]) underwent magnetic resonance spectroscopy at 3 Tesla. SCD and MCI participants were grouped together to form a single high-AD risk group (*N* = 24) for the purposes of statistical analyses. Partial correlations of GABA+/Cr level with verbal memory, assessed on California Verbal Learning Test-II, and nonverbal memory, assessed on Brief Visuospatial Memory Test and Rey-Osterrieth test, were examined separately within the high-AD risk and CN groups. GABA+/Cr levels were positively correlated with long-delayed verbal memory (*r* = 0.69, *P* = 0.009) and immediate nonverbal memory (*r* = 0.97, *P* = 0.03) in high-AD risk, but not in CN participants. These results remained significant after controlling for depression. These preliminary findings, which require replication due to the limited sample sizes, are the first report of an association between GABA+/Cr levels within the DM/DA-PFC and memory performance in high-AD risk individuals.

## Introduction

Alzheimer’s disease (AD) is the most common age-related neurodegenerative disorder, affecting ~24 million of the elderly population globally (Mayeux and Stern 2012). Around 1.3 million older adults over the age of 65 in the United States of America are projected to be diagnosed with AD by 2050 (Hebert et al. 2013). AD-related changes in the brain may occur 10–20 years prior to the diagnosis; thus, it is critical that we identify biomarkers for AD in its earliest stages (Jack et al. 2010).

Animal models of AD suggest that synaptic dysfunction due to the accumulation of amyloid β (Aβ) oligomers precedes neuronal loss (Selkoe 2002; Palop and Mucke 2010). Of the 2 major types of synapses in the central nervous system, glutamatergic and γ-aminobutyric acid (GABA)-ergic, which generate excitatory and inhibitory outputs, respectively, there is abundant evidence of impaired glutamatergic system in the pathogenesis of AD (Benarroch 2018). However, emerging data also support the contribution of GABAergic remodeling in the early stages of AD progression (Calvo-Flores Guzmán et al. 2018).

GABA is widely distributed in the brain and binds to ionotropic or metabotropic receptors on postsynaptic neurons to inhibit their function (Govindpani et al. 2017). As the primary inhibitory neurotransmitter in the brain, GABA plays a critical role in regulating responsiveness and excitability within neural networks and in synchronizing cortical neuronal signaling activity (Govindpani et al. 2017). Thus, GABA is involved in a wide range of physiological and biochemical processes, including the regulation of cognition, memory and learning, neural development, and adult neurogenesis (Govindpani et al. 2017). While GABAergic dysfunction has long been implicated in neuropsychiatric conditions such as epilepsy, mood disorders, autism, and schizophrenia (Verhoeff et al. 1999; Luscher et al. 2011; Fatemi and Folsom 2015), alterations of GABAergic circuits, whether as a primary event or as a compensatory response to excitotoxicity, also appear to contribute to AD progression by disrupting overall network function (Sailasuta et al. 2012; Huang et al. 2017). The GABAergic system has been recently identified as a potential target for AD intervention (Luo et al. 2015; Li et al. 2016; Govindpani et al. 2017). Preclinical studies suggest that GABA-A receptor agonists may have a neuroprotective effect, while antagonists enhance cognition, but these findings need to be corroborated using large clinical trials (Calvo-Flores Guzmán et al. 2018).

Other data show that cellular expression of GABA receptors may also be altered in AD. While there is an overall trend toward enhanced expression of the GABA-A receptor in the CA3 region, the findings are more complex within the CA1 region, with increased cellular expression in some subunits and decreased expression in others (Kwakowsky et al. 2018). Relevant to the current study, GABA clearance is also impaired in the AD brain (Fuhrer et al. 2017).

In addition, there is evidence of reduced GABA within temporal, medial frontal and parietal cortices in AD (Gueli and Taibi 2013; Bai et al. 2015). GABA level within the frontal lobes also decreases with age, cognition, and cortical volume (Porges, Woods, Edden, et al. 2017a). Furthermore, a study of healthy aging showed an association between GABA level within the dorsal anterior cingulate and executive functions (Marenco et al. 2018), suggesting that GABA within the dorsal anterior cingulate may be particularly sensitive to cognitive ability. This region overlaps with the prefrontal regions that show reduced GABA in major depressive disorder, which include the dorsomedial prefrontal cortex (dmPFC) in addition to dorsal anterior cingulate cortex (Hasler et al. 2007). Executive dysfunction and neuropsychiatric symptoms such as depression and anxiety may be early changes in AD (Geda et al. 2006; Guild et al. 2014; Mah et al. 2015). Taken together, these findings suggest that GABA level within the dorsomedial and dorsoanterolateral PFC regions may be associated with both cognition and mood.

In the current study, we measured GABA levels relative to Creatine (Cr) (GABA+/Cr) within the dorsomedial/dorsoanterolateral prefrontal cortex (DM/DA-PFC) in older adults at risk for developing AD and in a cognitively normal (CN) group using Magnetic Resonance Spectroscopy (MRS). The high-AD risk group included older adults with mild cognitive impairment (MCI), a prodrome of AD (Petersen 2004), and older adults with subjective cognitive decline (SCD), currently conceptualized as a preclinical stage of AD (Jessen et al. 2014). We assessed the relationship between DM/DA-PFC GABA+/Cr and memory within the high-AD risk group and within the CN group. We hypothesized that GABA+/Cr would be positively associated with verbal and nonverbal memory in high-AD risk participants, with weaker relationships in the CN group.

## Materials and Methods

### Participants

62 older adults (27 CN, 18 SCD, and 17 MCI) were scanned using MRS. Data from 24 participants (13 CN, 7 SCD, and 4 MCI) were excluded from the final analysis due to inability to obtain postprocessing fitting or poor spectral quality of MRS data due to movement artifacts. Thus, the final sample included 38 participants, which consisted of 14 CN (9 females, mean age = 68.3 years, SD = 6.4); 11 SCD (6 females, mean age = 75.1, SD = 6.6), and 13 MCI (5 females, mean age = 74.3, SD = 7.4). Due to their small sample sizes, SCD and MCI participants were grouped together to form a single group of high-AD risk participants (*N* = 24) for the purposes of statistical analyses.

All participants were aged 60 years or older with memory concerns, English language proficiency adequate for neuropsychological assessment, free of neurologic or psychiatric illnesses that may account for memory problems (e.g., previous stroke, Parkinson’s disease, depression, and dementia), and free of current psychotropic or memory enhancing medications within the past 3 weeks. Both CN and SCD participants scored within the normal range for age and gender on a neuropsychological battery. SCD participants had subjective memory concerns as defined by their responses on the self-report Memory Functioning Questionnaire- Frequency of Forgetting subscale (MFQ-FF) (Gilewski et al. 1990), a measure of subjective memory ability. Thus, lower scores on the MFQ-FF reflect subjective memory concerns. The MFQ-FF was selected as a measure of subjective memory concern because lower scores are sensitive to Aβ burden on Positron Emission Tomography imaging (Chételat et al. 2013). SCD participants’ MFQ-FF scores fell below the median score of 182 in our larger study sample of 143 participants, while CN participants scored above the median of 182 on the MFQ-FF. MCI participants met the National Institute on Aging and Alzheimer’s Association criteria for MCI (Albert et al. 2011) as well as specific neuropsychological criteria of performance on 2 or more memory tests greater than 1.5 SD lower than expected relative to overall intellect (Troyer et al. 2016). The study was approved by the Research Ethics Board of Baycrest Health Sciences.

### Data Acquisition

**Figure 1:**
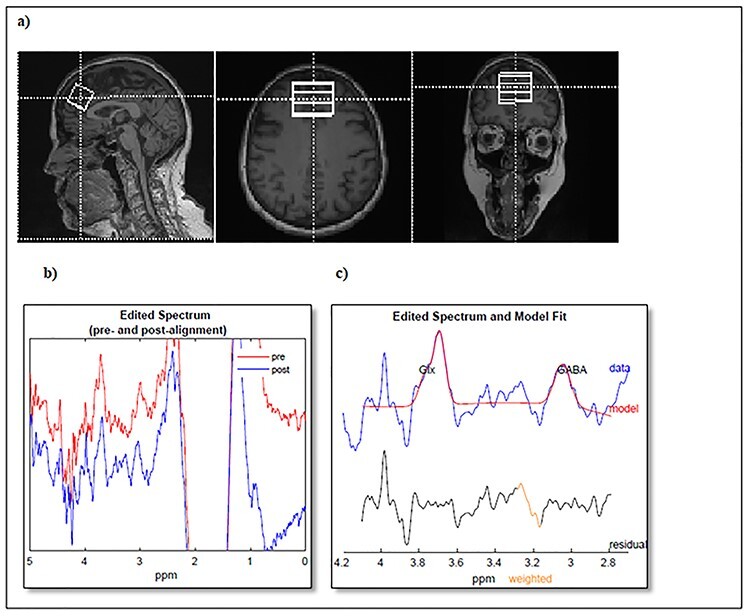
Data acquisition and processing. (*a*) Voxel placement of volume 25 × 40 × 25 mm^3^ in the dorsomedial/dorsoanterolateral prefrontal cortex ROI depicted by white solid boxes on the *T*_1_-MRI sagittal, axial, and coronal planes. (*b*) Gannet Load output of processed GABA-edited spectra with preprocessed spectrum prior to frequency and phase correction (shown in red), and postprocessed spectrum after frequency and phase correction (shown in blue). (*c*) Gannet Fit output of fitted GABA signal with GABA-edited difference spectrum (shown in blue) with the overlapping model of best fit (shown in red) and residual between the spectrum and the model of fit (shown in black).

We utilized the noninvasive J-difference edited MEscher-GArwood-Point Resolved Spectroscopy (MEGA-PRESS) pulse sequence to measure GABA level (Mescher et al. 1998), which allows fitting of GABA+ signals despite the presence of stronger signals of overlapping metabolites (Mullins et al. 2014). Proton MRS spectra were acquired from our region of interest (ROI) DM/DA-PFC which extended a 25 × 40 × 25 mm^3^ voxel and included portions of Brodmann areas 9, 24, and 32, dorsal and pregenual anterior cingulate gyrus, and adjacent medial frontal gyrus (Hasler et al. 2007). This ROI was selected based on a study that showed reduced GABA within DM/DA-PFC in patients with major depressive disorder (Hasler et al. 2007). Figure 1*a* shows the localization of the voxel of dimensions 25 × 40 × 25 mm^3^ on our ROI in the axial, coronal, and sagittal planes through the center of the voxel in the superoinferior direction.

Images were acquired using a TIM Trio 3 T System (Siemens) employing a 12-channel head array coil transmission. MRS data were acquired using MEGA-PRESS sequence with the following parameters: TE = 68 ms, TR = 2600 ms, 32 averages, bandwidth = 1200 Hz, and total acquisition duration = ~ 4 min. GABA-edited spectra were obtained by acquiring editing pulses at 1.9 ppm (edit pulse ON) and 7.46 ppm (edit pulse OFF). The estimation of in vivo GABA level in specific brain regions using noninvasive MRS technology is challenging due to the overlapping signals by higher concentrated metabolites such as choline (Cho), Cr, glutamate-glutamine (Glx), and N-acetyl aspartate (NAA) (Gao and Barker 2014). MEGA-PRESS aids in the removal of overlapping metabolites, and hence, we can detect free GABA signal co-edited with macromolecules (MM), that is, GABA+ signal at 3 ppm (Edden et al. 2014). The estimation of absolute GABA levels is challenging due to GABA’s small quantity (1.3–1.9 mmol/kg) in brain. Notably, non–GABA-related factors, such as motion, can also influence GABA quantification. Thus, a normalization factor that is typically employed relative to Cr level or unsuppressed water signals at 3 ppm (Edden et al. 2014). We measured GABA levels relative to Cr signals as the GABA+/Cr levels have higher inter- and intra-individual reproducibility compared with GABA+ relative to water signals (Bogner et al. 2010).

### Data Processing and Analysis

Phase correction and spectral fitting of GABA+ signals were quantified by Matlab-R2018b-based Gannet v3.0 available at https://github.com/richardedden/Gannet3.0/archive/master.zip (Edden et al. 2014). This automated software gives output in 2 forms: GannetLoad and GannetFit. GannetLoad processes time-domain MRS data into the frequency-domain GABA edited spectrum in phase correction. GannetFit integrates the edited GABA+ signal at 3 ppm and provides the spectral fitting of GABA+ signal with reference to Cr. This fully automated software built on the MATLAB environment widens the accessibility and reduces the bias due to rater-dependent variances. Data with poor shimming quality due to subject motion or scanner drift were not processed by Gannet to yield the fitting output (Edden et al. 2014). GABA-edited spectra processed by Gannet were evaluated for their spectral quality based on the model of best fit overlying on the GABA+ spectrum as shown in Figure 1*c*.

*T*_1_ volumetric segmentations and cortical reconstructions were processed using FreeSurfer v6.0 available at http://surfer.nmr.mgh.harvard.edu (Fischl and Dale 2000). We extracted whole brain gray matter (GM), white matter (WM), and ventricular cerebrospinal fluid (CSF) volumes and included these as covariates in statistical analyses.

### Neuropsychological and Behavioral Assessments

Participants were assessed using a comprehensive neuropsychological test battery which included the California Verbal Learning Test-II (CVLT-II) as a verbal memory measure (Delis et al. 2000). Because the neuropsychological battery was subsequently revised to minimize participants’ fatigue, only a subset of the high-AD risk group (*n* = 9) completed the Brief Visuospatial Memory Test (BVMT) (Benedict 1997) and the Rey-Osterrieth Figure Drawing Test (Rey-O) (Goldstein and Naglieri 2011) as nonverbal memory measures.

Participants also completed the Geriatric Depression Scale (GDS) (Yesavage 1988), a validated depression scale for older adults, to characterize the sample and to be included as a covariate in statistical analyses.

### Statistical Analyses

Statistical analyses were performed using SPSS v24.0. Due to nonnormality of the distribution of GABA+/Cr levels as determined by the Shapiro–Wilk test, we transformed GABA+/Cr levels using a two-step transformation method (Templeton 2011). In the first step, GABA+/Cr levels were transformed into percentile ranks to yield uniformly distributed probabilities. In the second step, inverse-normal transformation was applied to the percentile ranks.

To assess correlations between GABA+/Cr and memory measures, we used partial correlation controlling for age and whole brain tissue volumes of WM, GM, and CSF (Pizzi et al. 2017). Since alterations in the GABAergic system are well-established in depression (Sanacora et al. 1999), we repeated partial correlation analyses to include GDS scores as a covariate along with age and whole brain tissue volumes to assess the possibility that depressive symptoms accounted for any associations between GABA+/Cr level and memory.

Correlations were performed separately for the high-AD risk group and the CN group. For both groups, we correlated DM/DA-PFC GABA+/Cr levels with 3 verbal memory measures: CVLT-II Immediate Recall, CVLT-II Short-Delayed Free Recall, and CVLT-II Long-Delayed Free Recall. To reduce the probability of Type I error due to multiple comparisons, we applied a Bonferroni correction to yield a threshold of *P* value <0.017 for statistical significance (0.05/3 = 0.017).

Due to the limited number of participants who were administered with nonverbal memory tests, exploratory analyses were performed to examine the relationship between GABA+/Cr level and nonverbal memory measures (BVMT-Immediate and Delayed Recall, and Rey-O Immediate Recall) in the high-AD risk group (*n* = 9). As an even smaller number of CN participants completed the nonverbal memory tests, we were unable to perform correlations between GABA+/Cr levels and nonverbal memory measures in the CN group. For these exploratory analyses, we set the threshold at *P* value <0.05.

## Results

### Demographics, Clinical Characteristics, and Metabolite Compositions

Demographics and clinical characteristics of CN participants and the high-AD risk group, as well as SCD and MCI participants separately, are presented in Table 1. Relative to the CN group, depressive symptomatology as measured by GDS, was significantly greater in the high-AD risk group, as well as in the SCD group alone, although total GDS scores were still within the normal range (0–4) (Table 1) (Greenberg 2012). Mean GABA+/Cr level within DM/DA-PFC did not differ between the high-AD risk and CN or among SCD, MCI, and CN groups (Table 1).

**Table 1:** Demographic details: Demographics and clinical characteristics, memory, and metabolite compositions in CN and the high-AD risk group, as well as SCD and MCI participants separately

Descriptive		Mean (standard deviation)		
	**CN**	**High-AD risk group**	**SCD**	**MCI**
	**Demographics and clinical details**			
Total sample size	14	24	11	13
Total number of females	9	11	6	5
Age in years	68.3 (6.4)	74.7 (6.9)^*^^*^ ^**,a**^	75.1 (6.6)	74.3 (7.4)
Formal education in years	17.3 (3.2)	16.6 (2.4)	16.7 (2.6)	16.5 (2.3)
Montreal Cognitive Assessment scores	27.3 (1.5)^*^^*^ ^**,c**^	25.7 (2.2)^*^ ^**,a**^	26.9 (1.9)^*^ ^**,c**^	24.7 (2.1)
GDS	0.6 (0.8)	1.8 (1.6)^*^^*^ ^**,a**^	2.3 (2.0)^*^^*^ ^**,a**^	1.5 (1.1)
	***Neuropsychological measures***			
California Verbal Learning Test-II (CVLT-II) Immediate recall scores	59.4 (8.8)^*^^*^^*^ ^**,b,c**^	40.8 (8.5)^*^^*^^*^ ^**,a**^	**45.8 (8.9)** ^^*^,c^	36.5 (5.5)
CVLT-II short delayed free recall scores	12.4 (2.4)	8.2 (2.9)^*^^*^^*^ ^**,a**^	9.4 (2.8)^*^ ^**,a**^	7.2 (2.6)^*^^*^^*^ ^**,a**^
CVLT-II Long delayed free recall scores	12.6 (2.8)	8.3 (3.0)^*^^*^^*^ ^**,a**^	9.7 (2.8)^*^ ^**,a**^	7.1 (2.7)^*^^*^^*^ ^**,a**^
	**Metabolite compositions**			
GABA+/Cr levels	0.064 (0.04)	0.074 (0.05)	0.074 (0.04)	0.074 (0.06)

The details of SCD and MCI participants are present both individually and together as a group called high-AD risk. Data are presented as mean and standard deviations in brackets (). Single asterisk^*^ represents *P* value <0.05, double asterisks^*^^*^ represent *P* value <0.01, and triple asterisks^*^^*^^*^ represents *P* value <0.001 for pairwise comparisons.

^a^Represents data significantly different from CN.

^b^Represents data significantly different from SCD.

^c^Represents data significantly different from MCI. One-way ANOVA with Bonferroni correction was applied to compare data between CN, SCD, and MCI participants. Independent *t* test with two-tailed equal variances was applied to compare data between CN and high-AD risk group which is a combination of SCD and MCI participants. CN and high-AD risk groups are shaded as they are the groups compared in statistical analyses.

### Correlations between GABA+/Cr Level and Memory

#### High-AD Risk Group

Partial correlations between GABA+/Cr level and verbal memory controlling for age and GM, WM, and CSF volumes showed a robust correlation between GABA+/Cr and long-delayed free recall on the CVLT-II (*r* = 0.70, *P* = 0.005; Table 2a). Similarly, GABA+/Cr was significantly correlated with CVLT-II short-delayed free recall scores (*r* = 0.64, *P* = 0.01). A moderate correlation was observed between GABA+/Cr and immediate recall on the CVLT-II (*r* = 0.60, *P* = 0.02) but was not statistically significant at the Bonferroni-adjusted threshold of *P* value <0.017 (Table 2a).

**Table 2a:** Correlation analyses: Partial correlation between normally distributed GABA+/Cr levels within dorsomedial/dorsoanterolateral prefrontal cortex and verbal memory scores in CN (*N* = 14) and high-AD risk group (*N* = 24)

Scales	Subscales	Pearson’s *r* (significance level)	
Covariates: Age and brain tissue volumes (WM, GM, and CSF)		**CN**	**High-AD risk**
California Verbal Learning Test–II (CVLT-II)	Immediate recall	0.26 (0.5)	0.60 (0.02^*^)
	Long delayed free recall	−0.24 (0.5)	**0.70** (**0**.005^*^^*^)
	Short delayed free recall	−0.25 (0.5)	0.64 (0.01^*^^*^)
Covariates: Age, depression and brain tissue volumes (WM, GM, and CSF)		**CN**	**High-AD risk**
California Verbal Learning Test–II (CVLT-II)	Immediate recall	0.40 (0.3)	0.57 (0.04^*^)
	Long delayed free recall	−0.18 (0.7)	0.69 (0.009^*^^*^)
	Short delayed free recall	−0.21 (0.6)	0.62 (0.02^*^)

The correlation analyses are repeated with depression scores as a covariate in addition to age and whole brain tissue volumes. The Bonferroni-adjusted significance level *P* value <0.017 is applied here. Bonferroni-adjusted significance levels (*P* value < 0.017) are represented with double asterisks^*^^*^ and bold format. Significance levels (*P* value < 0.05) are represented with single asterisk^*^.

**Figure 2:**
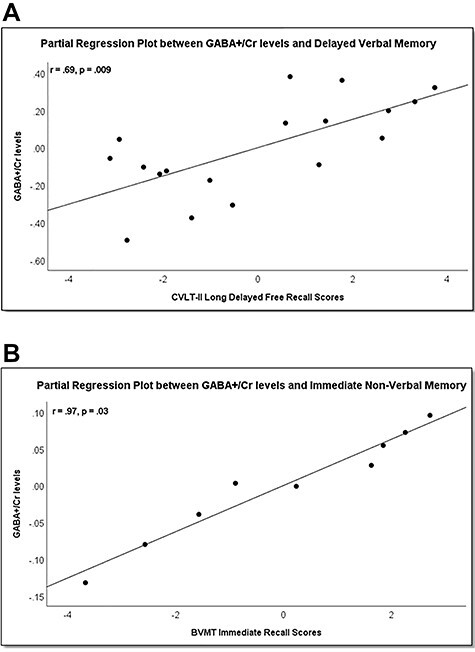
(*A*) Partial regression plot between GABA+/Cr levels and verbal memory. Higher GABA+/Cr levels were associated with better verbal memory performance as measured by CVLT-II Long-Delayed Free Recall in the total sample of high-AD risk participants (*n* = 24). GABA+/Cr levels are plotted against CVLT-II Long-Delayed Free Recall scores with age, depression, and brain tissue volumes (GM, WM, and CSF) as covariates. The straight line depicts the line of fit demonstrating the strength of this correlation (*r* = 0.69, *P* = 0.009). (*B*) Partial regression plot between GABA+/Cr levels and nonverbal memory. Higher GABA+/Cr levels were also associated with better nonverbal memory performance as measured by BVMT Immediate Recall in the subset of high-AD risk participants (*n* = 9) who completed these tests. GABA+/Cr levels are plotted against BVMT Immediate Recall scores with age, depression, and brain tissue volumes (GM, WM, and CSF) as covariates. The straight line depicts the line of fit demonstrating the strength of this correlation (*r* = 0.97, *P* = 0.03).

Partial correlations between GABA+/Cr level and verbal memory controlling for GDS in addition to age, GM, WM, and CSF volumes showed that the correlation between GABA+/Cr and CVLT-II Long-Delayed Free Recall remained statistically significant (*r* = 0.69, *P* = 0.009; Table 2a, Fig. 2*A*). Although short-delayed and immediate recall scores on the CVLT-II were moderately correlated with GABA+/Cr level when GDS was covaried (*r* = 0.62, *P* = 0.02; *r* = 0.57, *P* = 0.04, respectively) as shown in Table 2a, these correlation coefficients were not statistically significant at the Bonferroni-adjusted threshold of *P* value <0.017.

Exploratory analyses in the subset of our sample who completed nonverbal memory tests showed a strong correlation between GABA+/Cr and immediate recall scores on the BVMT (*r* = 0.99, *P* = 0.002) after controlling for age, GM, WM, and CSF volumes (Table 2b). The strength of the correlation remained unchanged when GDS was included as a covariate (*r* = 0.97, *P* = 0.03; Table 2b, Fig. 2*B*). Strong correlations with *r*’s >0.8 were similarly found between GABA+/Cr level and BVMT Delayed Recall and Rey-O Immediate Recall, in partial correlations analysis with age and GM, WM, and CSF volumes, with or without GDS, included as covariates. However, these findings were not statistically significant at the *P* value <0.05 threshold.

**Table 2b:** Correlation analyses: Partial correlation between normally distributed GABA+/Cr levels within dorsomedial/dorsoanterolateral prefrontal cortex and nonverbal memory scores in the subset of high-AD risk participants who completed the nonverbal memory measures (*N* = 9)

Scale	Subscale	Pearson’s *r* (significance level)
Covariates: Age and brain tissue volumes (WM, GM, and CSF)		High-AD risk
BVMT	Immediate recall	0.99 (0.002^*^^*^)
	Delayed recall	0.83 (0.08)
Rey-Osterrieth	Immediate recall	0.86 (0.06)
Covariates: Age, depression, and brain tissue volumes (WM, GM, and CSF)		High-AD risk
BVMT	Immediate recall	0.97 (0.03^*^)
	Delayed recall	0.79 (0.21)
Rey-Osterrieth	Immediate recall	0.87 (0.13)

The correlation analyses are repeated with depression scores as a covariate in addition to age and whole brain tissue volumes. The number of CN participants who completed these tasks was too small to perform similar partial correlations. Significance level *P* value <0.05 is applied to these exploratory analyses. Significance levels (*P* value < 0.01) are represented with double asterisks^*^^*^ and bold format. Significance levels (*P* value < 0.05) are represented with single asterisk^*^ and bold format.

#### CN Group

No significant correlations were observed between GABA+/Cr levels and verbal memory in the CN group (Table 2a). As only 6 CN individuals completed the nonverbal measures, we were unable to perform partial correlations between GABA+/Cr and nonverbal memory.

## Discussion

The purpose of our study was to assess the relationship between the GABA+/Cr level within DM/DA-PFC and memory in a sample of 24 high-AD risk and 14 CN participants. Although the limitation of sample size must be considered in interpreting the findings, our hypotheses were partially supported. Individuals in the high-AD risk group with higher GABA+/Cr level had greater long-delayed verbal recall, and this association remained statistically significant even after controlling for depression in addition to age and whole brain tissue volumes. Similar but less robust, associations were observed between GABA+/Cr level and CVLT-II immediate recall and short-delayed verbal recall scores. We also found evidence of strong positive associations between GABA+/Cr level and nonverbal memory, with a statistically significant correlation found for immediate recall on the BVMT-only. These associations appear to be specific to older adults at high-AD risk since similar findings were not observed in the CN group, in which no significant correlations were detected between GABA+/Cr level and memory measures. To the best of our knowledge, the current study is the first to report a strong association between DM/DA-PFC GABA+/Cr level and memory in older adults at high-AD risk.

Previous studies in CN older adults have linked enhanced excitatory glutamate/glutamine (Glx) levels in hippocampus with better verbal memory processes (Nikolova et al. 2017) and reduced inhibitory GABA levels in frontal cortex with cognitive decline (Porges, Woods, Edden, et al. 2017a). Our findings of an association between GABA+/Cr within DM/DA-PFC and memory in the high-AD risk group are consistent with these earlier studies. The present findings also extend the extant literature regarding reduced GABA in AD by demonstrating that the reduction in GABA appears to be tightly connected to verbal and nonverbal memory. This is in line with the established link between GABAergic and cholinergic systems. The GABAergic system stimulates the cholinergic neuronal system which is disrupted by AD pathology (Cummings and Back 1998), and it has been suggested that alterations in GABA level may represent a compensatory response to disruption of cholinergic networks in AD.

A major strength of our study is that participants were well-characterized with respect to cognition and mood, and none had a previous or current psychiatric disorder. This is an important methodological detail because alterations in GABA levels are found in depression, anxiety, and other psychiatric disorders (Kalueff and Nutt 2007; Luscher et al. 2011). Thus, the findings in our sample of high-AD risk participants are not likely to be confounded by underlying current or remitted psychiatric illness. A second strength of our study is that our statistical approach considered the impact of the whole brain tissue volumes, including GM, WM, and CSF on the GABA+/Cr level to reduce potential inter- and intra-individual variations of tissue components based on brain segmentation (Porges, Woods, Lamb, et al. 2017b). This is important because metabolite levels are known to vary across different brain tissues with higher distribution of GABA in GM compared with WM (Porges, Woods, Lamb, et al. 2017b).

Our study is not without limitations. As highlighted earlier, the main limitation is the small sample size, a consequence of the number of scans excluded from analysis due to movement artifacts, and hence poor spectral quality in both the high AD-risk (11 out of 35 scans) and CN groups (13 out of 27 scans). Thus, the extent to which our findings are stable and generalizable is unclear. The small sample sizes may have also precluded detection of statistically significant correlations in the CN group, and for some of the findings in the AD-risk group, where moderate-to-high correlation coefficients were observed (*r*’s in the range of 0.3–0.7) but not statistically significant. Due to the small numbers of SCD and MCI participants, both were combined into a single high-AD risk group for the purposes of data analyses. Thus, we are unable to establish the specificity of our findings to SCD or MCI. Finally, there are methodological limitations with the scanning parameters we used to acquire MRS images. Our scans included 32 averages, which is the minimum number of averages recommended by guidelines published after we had begun acquiring MRS data (Mikkelsen et al. 2018) but clearly does not meet current 2020 guidelines which specify a minimum of 240 averages (Peek et al. 2020). In addition, our acquisition time of ~4 min falls short of the recommended 7–10 min (Mullins et al. 2014). This shorter acquisition time was selected to optimize both power and the feasibility of older adults remaining as still as possible for the duration of the scan. This was a challenge for older adults with lower back pain or other pain syndromes and is reflected in the number of unusable images due to movement artifact.

Nevertheless, we report novel findings that show a direct relationship between GABA+/Cr level in the DM/DA-PFC and delayed verbal memory in high-AD risk individuals. Given that delayed verbal memory is a robust predictor of cognitive decline progressing from MCI to AD (Rabin et al. 2009), it is noteworthy that the strongest association observed was between GABA+/Cr level and CVLT-II long-delayed recall scores. These findings extend the literature reporting reduced GABA overall in AD by demonstrating that GABA level is closely linked to cognitive biomarkers of AD. Although the current results need to be replicated using larger sample sizes, our findings provide support for further investigations of the GABAergic system to discover novel biomarkers and interventions for AD.

## References

[ref1] Albert MS , DeKosky ST, Dickson D, Dubois B, Feldman HH, Fox NC, Gamst A, Holtzman DM, Jagust WJ, Petersen RC, et al. 2011. The diagnosis of mild cognitive impairment due to Alzheimer's disease: recommendations from the National Institute on Aging-Alzheimer's Association workgroups on diagnostic guidelines for Alzheimer's disease. Alzheimers Dement. 7:270–279. 21514249 10.1016/j.jalz.2011.03.008 PMC3312027

[ref2] Bai X , Edden RA, Gao F, Wang G, Wu L, Zhao B, Wang M, Chan Q, Chen W, Barker PB. 2015. Decreased γ-aminobutyric acid levels in the parietal region of patients with Alzheimer's disease. J Magn Reson Imaging. 41:1326–1331. 24863149 10.1002/jmri.24665 PMC5512098

[ref3] Benarroch EE . 2018. Glutamatergic synaptic plasticity and dysfunction in Alzheimer disease: emerging mechanisms. Neurology. 91:125–1232. 29898976 10.1212/WNL.0000000000005807

[ref4] Benedict RH . 1997. Brief visuospatial memory test–revised. Lutz, FL: Psychological Assessment Resources, Inc.

[ref5] Bogner W , Gruber S, Doelken M, Stadlbauer A, Ganslandt O, Boettcher U, Trattnig S, Doerfler A, Stefan H, Hammen T. 2010. In vivo quantification of intracerebral GABA by single-voxel 1H-MRS—how reproducible are the results? Eur J Radiol. 73:526–531. 19201120 10.1016/j.ejrad.2009.01.014

[ref6] Calvo-Flores Guzmán B , Vinnakota C, Govindpani K, Waldvogel HJ, Faull RL, Kwakowsky A. 2018. The GABAergic system as a therapeutic target for Alzheimer's disease. J Neurochem. 146:649–669. 29645219 10.1111/jnc.14345

[ref7] Chételat G , La Joie R, Villain N, Perrotin A, de La Sayette V, Eustache F, Vandenberghe R. 2013. Amyloid imaging in cognitively normal individuals, at-risk populations and preclinical Alzheimer's disease. NeuroImage: Clinical. 2:356–365. 24179789 10.1016/j.nicl.2013.02.006 PMC3777672

[ref8] Cummings JL , Back C. 1998. The cholinergic hypothesis of neuropsychiatric symptoms in Alzheimer's disease. Am J Geriatr Psychiatry. 6:S64–S78. 9581223 10.1097/00019442-199821001-00009

[ref9] Delis DC , Kramer JH, Kaplan E, Ober BA. 2000. California verbal learning test–second edition (CVLT-II). San Antonio, TX: The Psychological Corporation.

[ref10] Edden RA , Puts NA, Harris AD, Barker PB, Evans CJ. 2014. Gannet: a batch-processing tool for the quantitative analysis of gamma-aminobutyric acid–edited MR spectroscopy spectra. J Magn Reson Imaging. 40:1445–1452. 25548816 10.1002/jmri.24478 PMC4280680

[ref11] Fatemi SH , Folsom TD. 2015. GABA receptor subunit distribution and FMRP–mGluR5 signaling abnormalities in the cerebellum of subjects with schizophrenia, mood disorders, and autism. Schizophr Res. 167:42–56. 25432637 10.1016/j.schres.2014.10.010 PMC5301472

[ref12] Fischl B , Dale AM. 2000. Measuring the thickness of the human cerebral cortex from magnetic resonance images. Proc Natl Acad Sci. 97:11050–11055. 10984517 10.1073/pnas.200033797 PMC27146

[ref13] Fuhrer TE , Palpagama TH, Waldvogel HJ, Synek BJL, Turner C, Faull RL, Kwakowsky A. 2017. Impaired expression of GABA transporters in the human Alzheimer’s disease hippocampus, subiculum, entorhinal cortex and superior temporal gyrus. Neuroscience. 351:108–118. 28385633 10.1016/j.neuroscience.2017.03.041

[ref14] Gao F , Barker PB. 2014. Various MRS application tools for Alzheimer disease and mild cognitive impairment. Am J Neuroradiol. 35:S4–S11. 24742809 10.3174/ajnr.A3944 PMC4401041

[ref15] Geda YE , Knopman DS, Mrazek DA, Jicha GA, Smith GE, Negash S, Boeve BF, Ivnik RJ, Petersen RC, Pankratz VS. 2006. Depression, apolipoprotein E genotype, and the incidence of mild cognitive impairment: a prospective cohort study. Arch Neurol. 63:435–440. 16533972 10.1001/archneur.63.3.435

[ref16] Gilewski MJ , Zelinski EM, Schaie KW. 1990. The memory functioning questionnaire for assessment of memory complaints in adulthood and old age. Psychol Aging. 5:482. 2278670 10.1037//0882-7974.5.4.482

[ref17] Goldstein S , Naglieri JA. 2011. Encyclopedia of child behavior and development. New York: Springer.

[ref18] Govindpani K , Calvo-Flores Guzmán B, Vinnakota C, Waldvogel HJ, Faull RL, Kwakowsky A. 2017. Towards a better understanding of GABAergic remodeling in Alzheimer’s disease. Int J Mol Sci. 18:1813. 10.3390/ijms18081813 PMC5578199 28825683

[ref19] Greenberg SA . 2012. The geriatric depression scale (GDS). Best Practices in Nursing Care to Older Adults. 4:1–2.

[ref20] Gueli MC , Taibi G. 2013. Alzheimer’s disease: amino acid levels and brain metabolic status. Neurol Sci. 34:1575–1579. 23354600 10.1007/s10072-013-1289-9

[ref21] Guild EB , Vasquez BP, Maione AM, Mah L, Ween J, Anderson ND. 2014. Dynamic working memory performance in individuals with single-domain amnestic mild cognitive impairment. J Clin Exp Neuropsychol. 36:751–760. 25175752 10.1080/13803395.2014.941790

[ref22] Hasler G , van der Veen JW, Tumonis T, Meyers N, Shen J, Drevets WC. 2007. Reduced prefrontal glutamate/glutamine and γ-aminobutyric acid levels in major depression determined using proton magnetic resonance spectroscopy. Arch Gen Psychiatry. 64:193–200. 17283286 10.1001/archpsyc.64.2.193

[ref23] Hebert LE , Weuve J, Scherr PA, Evans DA. 2013. Alzheimer disease in the United States (2010–2050) estimated using the 2010 census. Neurology. 80:1778–1783. 23390181 10.1212/WNL.0b013e31828726f5 PMC3719424

[ref24] Huang D , Liu D, Yin J, Qian T, Shrestha S, Ni H. 2017. Glutamate-glutamine and GABA in brain of normal aged and patients with cognitive impairment. Eur Radiol. 27:2698–2705. 27966041 10.1007/s00330-016-4669-8

[ref25] Jack CR Jr , Knopman DS, Jagust WJ, Shaw LM, Aisen PS, Weiner MW, Petersen RC, Trojanowski JQ. 2010. Hypothetical model of dynamic biomarkers of the Alzheimer's pathological cascade. The Lancet Neurology. 9:119–128. 20083042 10.1016/S1474-4422(09)70299-6 PMC2819840

[ref26] Jessen F , Amariglio RE, Van Boxtel M, Breteler M, Ceccaldi M, Chételat G, Dubois B, Dufouil C, Ellis KA, Van Der Flier WM. 2014. A conceptual framework for research on subjective cognitive decline in preclinical Alzheimer's disease. Alzheimers Dement. 10:844–852. 24798886 10.1016/j.jalz.2014.01.001 PMC4317324

[ref27] Kalueff AV , Nutt DJ. 2007. Role of GABA in anxiety and depression. Depress Anxiety. 24:495–517. 17117412 10.1002/da.20262

[ref28] Kwakowsky A , Calvo-Flores Guzmán B, Pandya M, Turner C, Waldvogel HJ, Faull RL. 2018. GABAA receptor subunit expression changes in the human Alzheimer's disease hippocampus, subiculum, entorhinal cortex and superior temporal gyrus. J Neurochem. 145:374–392. 29485232 10.1111/jnc.14325

[ref29] Li Y , Sun H, Chen Z, Xu H, Bu G, Zheng H. 2016. Implications of GABAergic neurotransmission in Alzheimer’s disease. Front Aging Neurosci. 8:31. 26941642 10.3389/fnagi.2016.00031 PMC4763334

[ref30] Luo J , Lee SH, VandeVrede L, Qin Z, Piyankarage S, Tavassoli E, Asghodom RT, Aissa MB, Fà M, Arancio O. 2015. Re-engineering a neuroprotective, clinical drug as a procognitive agent with high in vivo potency and with GABA a potentiating activity for use in dementia. BMC Neurosci. 16:67. 26480871 10.1186/s12868-015-0208-9 PMC4612403

[ref31] Luscher B , Shen Q, Sahir N. 2011. The GABAergic deficit hypothesis of major depressive disorder. Mol Psychiatry. 16:383. 21079608 10.1038/mp.2010.120 PMC3412149

[ref32] Mah L , Binns MA, Steffens DC, AsDN I. 2015. Anxiety symptoms in amnestic mild cognitive impairment are associated with medial temporal atrophy and predict conversion to Alzheimer disease. Am J Geriatr Psychiatry. 23:466–476. 25500120 10.1016/j.jagp.2014.10.005 PMC4390420

[ref33] Marenco S , Meyer C, van der Veen JW, Zhang Y, Kelly R, Shen J, Weinberger DR, Dickinson D, Berman KF. 2018. Role of gamma-amino-butyric acid in the dorsal anterior cingulate in age-associated changes in cognition. Neuropsychopharmacology. 43:2285–2291. 30050047 10.1038/s41386-018-0134-5 PMC6135795

[ref34] Mayeux R , Stern Y. 2012. Epidemiology of Alzheimer disease. Cold Spring Harb Perspect Med. 2:a006239. 22908189 10.1101/cshperspect.a006239 PMC3405821

[ref35] Mescher M , Merkle H, Kirsch J, Garwood M, Gruetter R. 1998. Simultaneous in vivo spectral editing and water suppression. NMR Biomed. 11:266–272. 9802468 10.1002/(sici)1099-1492(199810)11:6<266::aid-nbm530>3.0.co;2-j

[ref36] Mikkelsen M , Loo RS, Puts NAJ, Edden RAE, Harris AD. 2018. Designing GABA-edited magnetic resonance spectroscopy studies: considerations of scan duration, signal-to-noise ratio and sample size. J Neurosci Methods. 303:86–94. 29476871 10.1016/j.jneumeth.2018.02.012 PMC5940501

[ref37] Mullins PG , McGonigle DJ, O'Gorman RL, Puts NA, Vidyasagar R, Evans CJ, Edden RA. 2014. Current practice in the use of MEGA-PRESS spectroscopy for the detection of GABA. Neuroimage. 86:43–52. 23246994 10.1016/j.neuroimage.2012.12.004 PMC3825742

[ref38] Nikolova S , Stark SM, Stark CE. 2017. 3T hippocampal glutamate-glutamine complex reflects verbal memory decline in aging. Neurobiol Aging. 54:103–111. 28363111 10.1016/j.neurobiolaging.2017.01.026 PMC5401796

[ref39] Palop JJ , Mucke L. 2010. Amyloid-β–induced neuronal dysfunction in Alzheimer's disease: from synapses toward neural networks. Nat Neurosci. 13:812. 20581818 10.1038/nn.2583 PMC3072750

[ref40] Peek AL , Rebbeck T, Puts NA, Watson J, Aguila MR, Leaver AM. 2020. Brain GABA and glutamate levels across pain conditions: a systematic literature review and meta-analysis of 1H-MRS studies using the MRS-Q quality assessment tool. Neuroimage. 210:116532. 31958584 10.1016/j.neuroimage.2020.116532

[ref41] Petersen RC . 2004. Mild cognitive impairment as a diagnostic entity. J Intern Med. 256:183–194. 15324362 10.1111/j.1365-2796.2004.01388.x

[ref42] Pizzi SD , Chiacchiaretta P, Mantini D, Bubbico G, Ferretti A, Edden RA, Di Giulio C, Onofrj M, Bonanni L. 2017. Functional and neurochemical interactions within the amygdala–medial prefrontal cortex circuit and their relevance to emotional processing. Brain Struct Funct. 222:1267–1279. 27566606 10.1007/s00429-016-1276-z PMC5549263

[ref43] Porges EC , Woods AJ, Edden RA, Puts NA, Harris AD, Chen H, Garcia AM, Seider TR, Lamb DG, Williamson JB, et al. 2017a. Frontal gamma-aminobutyric acid concentrations are associated with cognitive performance in older adults. Biol Psychiatry Cogn Neurosci Neuroimaging. 2:38–44. 28217759 10.1016/j.bpsc.2016.06.004 PMC5312683

[ref44] Porges EC , Woods AJ, Lamb DG, Williamson JB, Cohen RA, Edden RA, Harris AD. 2017b. Impact of tissue correction strategy on GABA-edited MRS findings. Neuroimage. 162:249–256. 28882635 10.1016/j.neuroimage.2017.08.073 PMC5705271

[ref45] Rabin LA , Paré N, Saykin AJ, Brown MJ, Wishart HA, Flashman LA, Santulli RB. 2009. Differential memory test sensitivity for diagnosing amnestic mild cognitive impairment and predicting conversion to Alzheimer's disease. Aging Neuropsychol Cogn. 16:357–376. 10.1080/13825580902825220 PMC3114447 19353345

[ref46] Sailasuta N , Harris K, Noeske R. 2012. Reduced cerebral GABA in patients with amnestic mild cognitive impairment (aMCI) may predict progression to Alzheimer’s disease (AD). Proc Intl Soc Mag Reson Med. 20:1002.

[ref47] Sanacora G , Mason GF, Rothman DL, Behar KL, Hyder F, Petroff OA, Berman RM, Charney DS, Krystal JH. 1999. Reduced cortical γ-aminobutyric acid levels in depressed patients determined by proton magnetic resonance spectroscopy. Arch Gen Psychiatry. 56:1043–1047. 10565505 10.1001/archpsyc.56.11.1043

[ref48] Selkoe DJ . 2002. Alzheimer's disease is a synaptic failure. Science. 298:789–791. 12399581 10.1126/science.1074069

[ref49] Templeton GF . 2011. A two-step approach for transforming continuous variables to normal: implications and recommendations for IS research. Commun Assoc Inf Syst. 28:4.

[ref50] Troyer AK , Vandermorris S, Murphy KJ. 2016. Intraindividual variability in performance on associative memory tasks is elevated in amnestic mild cognitive impairment. Neuropsychologia. 90:110–116. 27297728 10.1016/j.neuropsychologia.2016.06.011

[ref51] Verhoeff NPL , Petroff OA, Hyder F, Zoghbi SS, Fujita M, Rajeevan N, Rothman DL, Seibyl JP, Mattson RH, Innis RB. 1999. Effects of vigabatrin on the GABAergic system as determined by [123I] iomazenil SPECT and GABA MRS. Epilepsia. 40:1433–1438. 10528940 10.1111/j.1528-1157.1999.tb02016.x

[ref52] Yesavage JA . 1988. Geriatric depression scale. Psychopharmacol Bull. 24:709–711. 3249773

